# Associations between adolescents’ physical activity behavior and their perceptions of parental, peer and teacher support

**DOI:** 10.1186/s13690-020-00490-3

**Published:** 2020-10-23

**Authors:** Beata Pluta, Agata Korcz, Jana Krzysztoszek, Michał Bronikowski, Małgorzata Bronikowska

**Affiliations:** 1 Department of Recreation, Poznan University of Physical Education, Królowej Jadwigi 27/39, 61-871, Poznan, Poland; 2 Department of Didactics of Physical Activity, Poznan University of Physical Education, Królowej Jadwigi 27/39, 61-871, Poznan, Poland

**Keywords:** Parental support, Teacher support, Peer support, Physical activity, Adolescent

## Abstract

**Background:**

The aim of this study was to examine adolescents’ perceptions of parental, teacher, and peer support for physical activity, and to examine the associations between these perceptions and physical activity behavior.

**Methods:**

The study included 902 secondary school students, who completed the Short Scale of Youth’s Social Support Assessment (SSYSS). The level of physical activity – more specifically, moderate to vigorous physical activity – was measured using a Physical Activity Screening Measure. The associations were examined by a five-step hierarchical regression. Body mass index (BMI) was defined using the LMS method, which summarizes the distribution of BMI by age and gender in terms of three curves, L (lambda), M (mu), and S (sigma), and was based on a nationally representative sample of Polish children and adolescents.

**Results:**

The study indicated a positive correlation between MVPA (moderate to vigorous physical activity) and three sources of social support; however, in the regression model, this explained only part of the variance. In the hierarchical multiple regression analysis, MVPA level was predicted by five variables involvement in sports training, teachers support, parents support, gender and BMI. The direct effects for teachers and parents support were similar. This demonstrates that both teachers’ and parents’ social support exert influence on adolescent MVPA.

**Conclusions:**

The findings from this research suggest that school-based interventions for adolescents should specifically take into consideration family, teachers, and peers as important sources of social support for general physical activity promotion that aims to increase its levels.

## Background

Physical inactivity is the fourth leading cause of global morbidity; the prevalence of inactivity among people 15 years of age and older in the world is 31% [1]. The World Health Organization (WHO) has shown that there has been an epidemic decrease in physical activity (PA) in children (and adults), leading to noncommunicable diseases (NCDs) including obesity, cardiovascular disease, and diabetes [2]. NCDs kill 41 million people each year, equivalent to 71% of all deaths globally [3]. Current PA guidelines for children and youth aged 5–17 years recommend at least 60 min of MVPA per day [2], but nationally, only a small proportion of youth meet these guidelines [4]. Moreover, in Europe, more than two-thirds of European youth can be categorized as insufficiently active [5]. However, as indicated in a study by Dumith et al. [6], although a PA decline among girls was greater at younger ages (9–12 years old), among boys it was greater at older ages (13–16 years old). According to Cheng et al. [7], differences in PA levels between adolescent females and males, and between younger and older adolescents, may be partially explained by differences in social support provided by parents and peers. Public health researchers have recommended research on the type of social support most conducive to PA in children and adolescents.

Social support is still a contentious and poorly defined concept, and it is a multi-faceted issue [8, 9]. The most common measures related to social support are social relationships based on reciprocity, availability, and reliability, such as those that ensure the use of emotional support resources and provide distractions from stressful factors or information [9]. Furthermore, the WHO defines social support as being both “emotional and practical support characterising good social relations” and a social determinant of health [10].

According to developmental theory, the social influence of parents is important to young children, and this influence shifts to peers when children become adolescents [11]. Acceptance or encouragement of PA by self-help groups can make a significant difference in the practical implementation of the PA –related behaviors.

The anticipated benefits of social support are substantiated by research that has repeatedly shown that sources of parental, teacher, and peer support are associated with a higher level of PA among adolescents [12, 13]. Within the social domain, social support has been associated with engagement in regular PA by adolescents.

Factors outside the school environment very strongly affect adolescent PA. Social support from parents serves as one of the primary influences of youth PA-related behaviors. Tabak et al. [14] described emotional support that consisted of passing on positive emotions reflecting care, instrumental support that encompassed providing tools and ways of behaving, and financial support.

According to the results of a study by Van Der Horst et al. [15], the relationship between social support and the level of participation in PA is strongly influenced by the age of the youthful. Adolescence is considered a period of increasing independence from parents and of closer relationships with peers, whose influence begins to outweigh that of parents [2]. Young people spend more of their time at school than in any other setting, and therefore, experiences at school, especially involving relationships with teachers and classmates, have a notable influence on adolescent development [16].

Study findings suggest that peer support plays a more important role than family support in adolescent PA [17]. Therefore, in that specific time of youth, family support, and even teacher support, may have less impact than peer support on adolescent PA levels. Furthermore, in a study by Campos et al. [18], social support was associated (among other variables) with levels of PA that indicated that the absence of high support from parents and friends might reduce the level of PA in adolescents. A study by Bokhorst et al. [16] showed that parents and friends were perceived to be equally supportive, and friends support exceeded parental support only for ages 16–18 years. Support from teachers was lower in the older age groups, and this was related to the transition from primary to secondary school. In the same study, the authors indicated that girls perceived more support from teachers, classmates, and friends than did boys [16].

Gender difference is another important factor that needs to be considered in understanding the source of social support (peers, parents, teachers) and its PA relationship. Boys and girls have different preferences for social support [19], and this may contribute to gender differences in the relationship between PA and various sources of social support. Therefore, identifying and comparing gender differences in this relationship could provide valuable information for designing gender-specific intervention strategies, not only in Poland [19].

Many studies have examined the influence of peers on their friends’ PA behaviors [20], and some of them, conducted among African American adolescents, found that boys received more peer support to engage in PA than did girls, and that girls often reported receiving no peer support for PA [13]. Based on other studies, it can be concluded that peer support during PA is associated with increased PA, and this association may be greater for overweight adolescents [1]. In a study by Bronikowski et al. [21], boys obtained more support from teachers, while in another study, girls reported greater social support in general [22].

The current study explores the associations between three sources of social support (parental support, peer support, and PE teacher support) and MVPA, gender, and BMI status (underweight, normal weight, and overweight) among adolescents from the province of Wielkopolska, Poland. The results of this study can contribute to a better understanding of factors associated with adolescent PA, and improve the knowledge necessary to design intervention programs to promote activity in secondary school students.

## Methods

The cross-sectional study included 902 secondary school boys (*N* = 405) and girls (*N* = 497) (aged 16.4 ± 0.7 years) from the province of Wielkopolska. The study participants were recruited from the same grade level at schools in three major cities of the province. Permission to carry out the research was secured separately and was granted by the principals of the institutions concerned. All respondents were informed that their participation would be anonymous, and were informed of the aim of the research and the manner in which the results would be used for scientific purposes. This research study was conducted by the diagnostic survey method with the use of a structured survey questionnaire that had previously been prepared, tested, and assessed by competent judges. Questionnaires were completed in whole-class groups during regular school lessons and took approximately 25 min to complete. Furthermore, the respondents were informed that they could abstain in cases where they did not wish to answer certain questions included in the questionnaire.

The study variables included three indicators of support (parents, teachers, and peers), a self-reported amount/level of PA (MVPA), BMI (according to Cole), and gender. In addition, the examined students were asked to answer question concerning their statues of involvement in sport (no involvement, amateur, and professional training). Professional participation meant engagement in regular system of competitions, organized by a sports federation, whereas amateurism meant taking part in sports as a hobby, for pleasure.

### Short scale of Youth’s social support assessment

The variables, which included parental, peer, and teacher support, were measured using the 18 item SSYSS. This self-report instrument was designed to measure the impact of parental (5 items), peer (8 items), and teacher (5 items) support, using a five-point Likert scale that allowed the respondent to express how much they agreed or disagreed with a particular statement, from 1 (strongly disagree) to 5 (strongly agree), with higher scores corresponding to higher agreement. The maximum total score was 25 points for the parental and teacher subscales, and 40 points for the peer subscale. The assessment covered all environments where a young person might live. This tool is a widely accepted, accurate, and valid measure of youth social support [23, 24]. SSYSS has satisfactory psychometric properties, and it is reliable and accurate. Its reliability was checked by estimating the internal compatibility of the tool (using Cronbach’s coefficient alpha). The relevance of the SSYSS was tested by considering its two aspects – i.e., its content relevance, using Lawshe’s CVR coefficient, and its theoretical validity – by conducting a factor analysis using an exploratory variation [25]. Internal consistency rates for the subscales of the SSYSS used in this study were established using Cronbach’s alpha test. For the teacher support scale, the alpha value was 0.84; for peer support, it was 0.86, and for parental support, it was 0.84.

To summarize, for all the subscales, the values of Cronbach’s alpha could be considered unquestionably satisfactory, as all of them exceeded 0.8. The critical values of the discriminant power coefficients at the level of α = 0. 05 were 0.39, 0.28, 0.20, and 0.14, respectively. Acceptable values of discriminatory power have, therefore, been obtained for all items.

The support from parents, teachers, and peers was categorized by using the scores of individuals, normalized to a sten scale [26]. Individuals with sten scores of 1–4 were classified as receiving a low level of support, those with 5–6 as having a medium level of support, and those with 7–10 as receiving a high level of support [27].

### Physical activity

MVPA was measured using a physical activity screening measure [28]. One of the reasons for using this measure was its ease of application in a school setting. Reliability was established at ICC = 0.77 and validity at 푟 = 0.40. This measure was used in an earlier population study in Poland [29]. Participants were asked to answer two questions: (Q1) Over the past 7 days, on how many days were you physically active for a total of at least 60 min per day?; (Q2) Over a typical or usual week, on how many days are you physically active for a total of at least 60 min per day? The MVPA index was calculated based on the following formula: MVPA = (Q1 + Q2)/2, where MVPA = PA index; Q1 is the number of physically active days during the past 7 days; Q2 is the number of physically active days during a typical (usual) week.

### BMI

BMI was calculated from adolescents’ self-reported height and weight. Growth references for height, weight, and BMI were constructed (separately for each sex) with the L, M, S method using data from a large, recent population-representative sample of school-aged children and adolescents in Poland without known disorders affecting growth [30, 31]. The prevalence of overweight, and obesity according to the International Obesity Task Force (IOTF) criteria was determined with the use of LMS Growth software [29]. Updated growth references for Polish school-aged children and adolescents were compared with Polish growth references from the 1980s, the Warsaw 1996–1999 reference, German, and 2000 Centers for Disease Control and Prevention (CDC) references. A Box-Cox power transformation was used to normalize the data at each age. Participants were classified as underweight, normal weight, or overweight according to age- and gender-specific cut-off points for children and adolescents [29, 32].

The personal characteristics of the secondary school students were documented and adequately protected in accordance with research ethics.

### Data analysis

All statistical analyses were completed using the STATISTICA ver. 13.1 software package (StatSoft, Krakow, Poland). The data were screened and checked against the assumptions of regression analysis. The Mann–Whitney test (Z), Kruskal-Wallis test (H), and Spearman’s rank correlation coefficients (r_s_) were used to assess the significance and power of the relationships between variables, with the value of correlation strength: ≤0.39 weak, 0.40–0.59 moderate and ≥ 0.60 strong [33]. To describe differences between groups, effect sizes were calculated as the difference between means divided by the pooled SD. Using Cohen’s criteria, an effect size ≥0.20 and < 0.50 was considered small, with ≥0.50 and < 0.80 being considered medium, and 0.80 considered large. A hierarchical multiple regression was conducted with MVPA score as a dependent variable. The model included five independent variables that correlated with MVPA.

The threshold of statistical significance for the inclusion of independent variables in the multiple regression models was set at *p* < 0.05.

## Results

The demographics of the study population are presented in Table 1, that the gender-specific descriptive statistics of the total sample are summarized. The sample was 55.1% female (*N* = 497) and 44.9% male (*N* = 405). Significant gender differences were found. Boys were more likely than girls to participate in PA, and there were significant gender differences in the recorded amount of MVPA (*Z* = 3.011, *p* = 0.003, d = 0.21). The greatest differences were observed for body height and body mass (*Z* = 20.759, *p* < 0.001, d = 1.84 and *Z* = 18.232, *p* < 0.001, d = 1.43, respectively) and the smallest difference was noted for parent support (*Z* = − 4.303, *p* < 0.001, d = 0.30).

**Table 1:** Demographic and psychosocial characteristic of the secondary school students

Variable	Girls (*N* = 497)	Boys (*N* = 405)	
	Mean ± SD		*p* value
age [years]	16.36 ± 0.73	16.41 ± 0.71	0.3492^a^
height [cm]	167.55 ± 6.41	180.17 ± 7.34	< 0.0001^a^
weight [kg]	56.95 ± 7.80	70.79 ± 11.59	< 0.0001^a^
MVPA [days/week]	3.47 ± 1.67	3.82 ± 1.71	0.0026^a^
teachers support [sten]	5.58 ± 1.98	5.80 ± 2.07	0.1017^a^
parents support [sten]	5.94 ± 2.03	5.33 ± 2.05	< 0.0001^a^
peers support [sten]	6.07 ± 2.43	5.90 ± 2.29	0.2930^a^
	% (N)		
BMI [Cole]			< 0.0001^b^
underweight	21% (106)	14% (58)	0.0063^c^
normal weight	64% (316)	56% (226)	0.0145^c^
overweight	15% (75)	30% (121)	< 0.0001^c^
involvment in sports training			0.8168^b^
no training	20% (97)	19% (78)	–
amateur training	54% (211)	52% (211)	–
professional training	27% (116)	29% (116)	–

BMI Body Mass Index values have been adjusted for age and gender, SD – standard deviation, ^*^significant difference between genders: *p* < .05, ^a^ Mann-Whitney U test (Z), ^b^ chi-square test, ^c^ post-hoc test

The coefficients of correlation between selected independent variables and MVPA are presented in Table 2. In generally, all variables connecting with different sources of the social support demonstrated correlation in the expected direction with MVPA. Parental support for PA is greater for boys than for girls. The mean recorded amount of MVPA increases with the frequency of parental, teacher, and peer support indicators. Although all bivariate correlations between the social support variables and MVPA are positive and statistically significant (*p* < 0.05), they are generally low (Table 2). The correlation between BMI (according to Cole) and social support was positive, but was not statistically significant. In turn, MVPA and BMI in categories differ significantly between adolescents (H (2,902) = 7.58, *p* = 0.022, d = 0.24). This important differentiation is influenced by distinction differences between underweight and overweight adolescents. The similar relationship in the examined group existed between MVPA and students who declared no participation in any form of sports, on an amateur level and were involved in sport within professionally organized structures (H (2,902) = 78,43 *p* ≤ 0.0001, d = 0.85).

**Table 2:** Correlation among study variables

variable	1	2	3	4	5	6
1. MVPA [days/week]	–					
2. teachers support (sten)	0.15*^a^	–				
3. parents support (sten)	0.12*^a^	0.20*^a^	–			
4. peers support (sten)	0.09*^a^	0.19*^a^	0.37*^a^	–		
5. BMI [Cole]	0.24*^b^	0.17^b^	0.07^b^	0.06^b^	–	
underweight vs normal weight	0.25*^b^					
underweight vs overweight						
normal weight vs overweight						
6. involvement in sports training	0.85*^b^	0.10^b^	0.19^b^	0.07^b^	1.18^c^	–
no training vs amateur training	0.57*^b^					
no training vs professional training	0.90*^b^					
amateur training vs professional training	0.31*^b^					
7. gender	3.01*^d^	1.64^d^	−4.30*^d^	−1.05^d^	30.73*^c^	0.40^c^
Mean	3.62	5.68	5.66	6.00		
SD	1.698	2.022	2.060	2.369		
range (min-max)	0–7	2–10	1–10	1–10		

* - *p* < 0.05, ^a^ Spearman’s R, ^b^ effect size (d), ^c^ chi-square test, ^d^ Mann-Whitney U test (Z)

Hierarchical multiple regression was conducted with MVPA level as a dependent variable (Table 3). The model included five independent variables that correlated significantly with MVPA.

**Table 3:** Predictors of MVPA in adolescents, identified on hierarchical regression analysis

variable	R^2^	β	F	*p* value
Step 1	7.8		76.58	≤0.0001
involvment in sports training		0.28		≤0.0001
Step 2	9.8		48.63	≤0.0001
involvment in sports training		0.27		≤0.0001
teachers support (sten)		0.14		≤0.0001
Step 3	10.6		35.44	≤0.0001
involvment in sports training		0.27		≤0.0001
teachers support (sten)		0.13		≤0.0001
gender		−0.09		0.0041
Step 4	11.4		29.00	≤0.0001
involvment in sports training		0.27		≤0.0001
teachers support (sten)		0.11		0.0005
gender		−0.11		0.0009
parents support (sten)		0.10		0.0032
Step 5	11.7		23.75	≤0.0001
involvment in sports training		0.27		≤0.0001
teachers support (sten)		0.11		0.0006
gender		−0.11		0.0005
parents support (sten)		0.10		0.0032
BMI		0.05		0.1086
*N* = 902				

In the first step, involvement in sport training was included in the model (*R*^*2*^ *= 7.8%, p ≤ 0.0001*). Then, teacher support was added (*R*^*2*^ *= 9.8%, p ≤ 0.0001*), followed by gender (*R*^*2*^ *= 10.6%, p ≤ 0.0001*). In the fourth step, parents support was added to the model. The final model was significant and explained a considerable proportion of variance in MVPA in adolescents (*R*^*2*^ *= 11.7%, p ≤ 0.0001*), although one of the components, BMI index, was not a significant predictor of the dependent variable (*p =* 0.1086). Hierarchical multiple regression with MVPA as a dependent variable showed that only a small amount of variance was explained. No other statistically significant associations were observed.

## Discussion

Social support can have a large impact on the level of PA undertaken. Information obtained from the study can be used to take action to increase the level of PA among teenagers. The majority of studies focus on total social support and modeling from parents, family, and friends. This present study provides evidence of the relationship between different types of support (from parental to teacher and peer support) for teenage girls and boys and their levels of PA. This research attempts to determine the causes of inactivity in this social group.

Identification of factors that mediate PA behaviors in adolescents is the key to the development of effective interventions to promote PA. Furthermore, exploring the association between BMI and social support is important, as some researchers have indicated that overweight adolescents may be more socially isolated [34]. Others have found that overweight children report lower levels of social support for PA [35].

Early support from parents, teachers, and peers has been reported as a significant predictor of self-reported engagement in PA [19]. Support from friends was found to be the most important social environmental factor for young adolescents, which may make sense from a developmental perspective, as peer support in adolescence is generally a dominant social factor that plays a critical role in the development of PA motivation and engagement [36].

Our study on young adolescents indicated a positive correlation between MVPA and social support; however, in the regression models examined, this correlation explained only a part of the variance. When five-steps hierarchical regression was performed it explained a considerable proportion of variance in MVPA in adolescents (R2 = 11.7%, *p* = 0.1086). Following regression predictors were included in that final model: involvement in sport training (β = 0.27; *p* ≤ 0.0001), PE teachers’ (β = 0.11; *p* = 0.0006) and parents’ (β = 0.10; *p* = 0.0032) support perceived by adolescents in regard to participation in leisure time PA (LTPA) from moderate to vigorous intensity. At the same time a significant negative relation (β = − 0.11; *p* = 0.0005) was found between MVPA and gender, which might suggest that in examined age group seems to be less influential factor. Obviously, there are other factors that might mediate these relationships, such as the school environment, sporting traditions of the local community or a particular family, individual interests, and engagement in sports [37]. Verloigne et al. [38] reported a positive correlation between parental support and MVPA both on weekdays and weekends, though it was mainly support in the form of logistic actions. Parental control and concern were both inversely related to MVPA on weekdays, while with adolescents, peer interest was positively related to MVPA on those days. The authors indicated that internal barriers (likes/dislikes, insufficient time, preferences, laziness) were the factors that significantly mediated the relationship between parental logistic support and MVPA on weekdays, which was not the case with external barriers (homework, nobody to play with, health issues, no conditions, lack of funds) or with self-efficacy. Kremers et al. [39] indicated that both parental and peer support influence the healthy choices and behaviors of adolescents via “automatic” pathways during spontaneous engagement in a particular behavior, and/or in the case of peers, through indirect personal and individual factors, and may be mediating factors in helping to overcome perceived barriers and logistical problems.

In another study on young, non-white adolescent girls, Larson et al. [40] found that lower PA among female friends was associated with higher BMI (infrequent family meals and an unsafe environment also contributed to BMI) and that the problem may lie at home. In our study, there was no relationship between BMI and any source of support. However, higher parental support was associated with higher MVPA, but only in boys, and not in adolescent girls. Interestingly, and unlike our research, a study by Yao et al. [41] suggested that both peers and parents have an influence on the PA, but they studied teenage girls.

Nevertheless, it seems that teachers and parents remain the most influential sources of support in terms of MVPA, at least until the age of young adolescence. Thus, these particular social groups (teachers and parents) should be strongly aware of PA and weight loss programs among youth, which ought to focus on parental role modeling. The effectiveness of such an approach was confirmed by Crawford et al. [42] in their research.

There are some limitations that need to be acknowledged. The current study was cross-sectional and did not examine other potentially influential factors (such as access to facilities, family sporting traditions and role modelling, and school policies), which might be considered a limitation. However, the reasonably large sample size and the similar cultural backgrounds of the examined adolescents (coming from urban areas of cities) seem to be a strength. Nevertheless, in further research, multi-contextual and longitudinal studies are needed to consider other potentially mediating variables, and the scale of the variance in youth MVPA could be replace with another tool measuring PA. Longitudinal research has the ability to make causal statements, but an experimental study would be more helpful in determining what affect an increase of PA would have in cultural and social context of development of young adolescents from Poland.

## Conclusion

There is positive relationship between three sources of social support (parents, teachers, and peers) and the level of PA undertaken by Polish adolescents in urban areas. To prevent any further decline in the level of PA of young people, a supportive environment around PA must be created. As a recommendation, we would like to point out that further school-based interventions and PE curricula-related program changes should target PE teachers and their modelling role, and peer norms, which, when combined, could enhance better nutrition and PA choices and behaviors among young adolescents.

The promotion of PA is of great importance for adolescents as it might lead to the development not only of favorable health behaviors, but also of better social interactions, which are so needed nowadays in youth.

## Data Availability

Please contact the corresponding author for data requests.

## Abbreviations

WHO: World Health Organization
PA: physical activity
MVPA: moderate to vigorous physical activity
BMI: Body Max Index
SSYSS: Short Scale of Youth’s Social Support Assessment
